# Supplementary material for: Effects of personal relevance and simulated darkness on the affective appraisal of a virtual environment
Source: PeerJ. 2016 Feb 25;4:e1743. doi: 10.7717/peerj.1743 (PMC4788201; doi:10.7717/peerj.1743)
Supplement: Data S1 [file peerj-04-1743-s001.zip › data/SPSS_codebook.docx]

# Benodigde gegevens SPSS / WASP bestand

| **DATA** | **VARIABLE** | **ARGUMENTS** | **Extra Info** |
| --- | --- | --- | --- |
| *Algemene informatie* | | | |
| RespondentID | ID | NUMBER {1 – 72} |  |
| Conditie | Condition | NUMBER {1 – 6} | 1 = Day  2 = Night  3 = Day/Day  4 = Night/Night  5 = Day/Night  6 = Night/Day |
| Time-of-day (IV) | Independent_VE | NUMBER {1 – 2} | 1 = Day  2 = Night |
| Task (IV) | Independent_TASK | NUMBER {1 – 3} | 0 = No task  1 = Same environment  2 = Contrary environment |
| ID in de conditie | Condition_ID | NUMBER {1 – 12} |  |
| Leeftijd | Age | TEXT |  |
| Opleiding | Degree | NUMBER {1 – 7} | 1 = Lager onderwijs  2 = vmbo  3 = havo  4 = vwo  5 = mbo  6 = hbo  7 = wo |
| *Orienation and navigation* | | | |
| Ik kan goed mijn weg vinden in een onbekende omgeving. | Onbekend | NUMBER {-3 – 3} | -3 = Helemaal mee oneens  0 = Neutraal  3 = Helemaal mee eens |
| Ik kan <i>'s nachts</i> goed mijn weg vinden in een bekende | BekendNacht | NUMBER {-3 – 3} |  |
| Ik kan <i>'s nachts</i> goed mijn weg vinden in een <i>onbekende</i> omgeving. | OnbekendNacht | NUMBER {-3 – 3} |  |
| Ik durf in mijn eentje overdag in een <i>onbekende</i> omgeving te lopen. |  | NUMBER {-3 – 3} |  |
| Ik durf in mijn eentje <i>'s nachts</i> in een <i>onbekende</i> omgeving te lopen. | OnbekendOverdagDurven | NUMBER {-3 – 3} |  |
| Ik kan me goed oriënteren in het donker. | OrienterenDonker | NUMBER {-3 – 3} |  |
| Ik kan me goed oriënteren bij daglicht. | OrienterenDaglicht | NUMBER {-3 – 3} |  |
| Ik voel me onprettig in het donker. | OnprettigDonker | NUMBER {-3 – 3} |  |
| Hoe vaak speelt u 3D computer spellen? | Games3D | NUMBER {1 – 5} | 1 = Nooit  2 = Heel soms  3 = Soms  4 = Vaak  5 = Heel vaak |
| Hoe vaak maakt u gebruik van andere virtuele omgevingen dan computer spellen (bv. Second Life)? | VirtualEnvironments | NUMBER {1 – 5} |  |
| *PANAS (1)* | | | |
| Bedroefd | BEDROEFD#1 | NUMBER {1 – 5} | **Negative affect (1)** |
| Terneergeslagen | TERNEERGESLAGEN#1 | NUMBER {1 – 5} |  |
| Schuldig | SCHULDIG#1 | NUMBER {1 – 5} |  |
| Angstig | ANGSTIG#1 | NUMBER {1 – 5} |  |
| Vijandig | VIJANDIG#1 | NUMBER {1 – 5} |  |
| Prikkelbaar | PRIKKELBAAR#1 | NUMBER {1 – 5} |  |
| Beschaamd | BESCHAAMD#1 | NUMBER {1 – 5} |  |
| Nerveus | NERVEUS#1 | NUMBER {1 – 5} |  |
| Rusteloos | RUSTELOOS#1 | NUMBER {1 – 5} |  |
| Bang | BANG#1 | NUMBER {1 – 5} |  |
| Geïnteresseerd | GEINTERESSEERD#1 | NUMBER {1 – 5} | **Positive affect (1)** |
| Opgewekt | OPGEWEKT#1 | NUMBER {1 – 5} |  |
| Sterk | STERK#1 | NUMBER {1 – 5} |  |
| Enthousiast | ENTHOUSIAST#1 | NUMBER {1 – 5} |  |
| Zelfverzekerd | ZELFVERZEKERD#1 | NUMBER {1 – 5} |  |
| Alert | ALERT#1 | NUMBER {1 – 5} |  |
| Geïnspireerd | GEINSPIREERD#1 | NUMBER {1 – 5} |  |
| Vastberaden | VASTBERADEN#1 | NUMBER {1 – 5} |  |
| Aandacht | AANDACHT#1 | NUMBER {1 – 5} |  |
| Energiek | ENERGIEK#1 | NUMBER {1 – 5} |  |
| Negatieve affect | NA#1 | NUMBER {10 – 50} |  |
| Positieve affect | PA#1 | NUMBER {10 – 50} |  |
| *SAM (1)* | | | |
| Pleasure | SAM#1Pleasure | NUMBER {1 – 9} |  |
| Arousal | SAM#1Arousal | NUMBER {1 – 9} |  |
| Dominance | SAM#1Dominance | NUMBER {1 – 9} |  |
|  |  |  |  |
| *Affective Quality* | | | |
| Behaaglijk | BEHAAGLIJK | NUMBER {1 – 7} | **Coziness** |
| Veilig | VEILIG | NUMBER {1 – 7} |  |
| Intiem | INTIEM | NUMBER {1 – 7} |  |
| Beangstigd | BEANGSTIGEND | NUMBER {1 – 7} | **Tenseness** |
| Bedreigend | BEDREIGEND | NUMBER {1 – 7} |  |
| Gespannen | GESPANNEN | NUMBER {1 – 7} |  |
| Inspirerend | INSPIREREND | NUMBER {1 – 7} | **Liveliness** |
| Levendig | LEVENDIG | NUMBER {1 – 7} |  |
| Stimulerend | STIMULEREND | NUMBER {1 – 7} |  |
| Zakelijk | ZAKELIJK | NUMBER {1 – 7} | **Detachment** |
| Formeel | FORMEELS | NUMBER {1 – 7} |  |
| Hoe zou u de sfeer van het gebied omschrijven? | SFEER | TEXT |  |
| Coziness | COZINESS | NUMBER {-3 – 3} |  |
| Liveliness | LIVELINESS | NUMBER {-3 – 3} |  |
| Tenseness | TENSENESS | NUMBER {-3 – 3} |  |
| Detachment | DETACHMENT | NUMBER {-3 – 3} |  |
| *SAM (2)* | | | |
| Pleasure | PLEASURE_2 | NUMBER {1 – 9} |  |
| Arousal | AROUSAL_2 | NUMBER {1 – 9} |  |
| Dominance | DOMINANCE_2 | NUMBER {1 – 9} |  |
|  |  |  |  |
| *PANAS (2)* | | | |
| Bedroefd (2) | BEDROEFD#2 | NUMBER {1 – 5} | **Negative affect (2)** |
| Terneergeslagen (2) | TERNEERGESLAGEN#2 | NUMBER {1 – 5} |  |
| Schuldig (2) | SCHULDIG#2 | NUMBER {1 – 5} |  |
| Angstig (2) | ANGSTIG#2 | NUMBER {1 – 5} |  |
| Vijandig (2) | VIJANDIG#2 | NUMBER {1 – 5} |  |
| Prikkelbaar (2) | PRIKKELBAAR#2 | NUMBER {1 – 5} |  |
| Beschaamd (2) | BESCHAAMD#2 | NUMBER {1 – 5} |  |
| Nerveus (2) | NERVEUS#2 | NUMBER {1 – 5} |  |
| Rusteloos (2) | RUSTELOOS#2 | NUMBER {1 – 5} |  |
| Bang (2) | BANG#2 | NUMBER {1 – 5} |  |
| Geïnteresseerd (2) | GEINTERESSEERD#2 | NUMBER {1 – 5} | **Positive affect (2)** |
| Opgewekt (2) | OPGEWEKT#2 | NUMBER {1 – 5} |  |
| Sterk (2) | STERK#2 | NUMBER {1 – 5} |  |
| Enthousiast (2) | ENTHOUSIAST#2 | NUMBER {1 – 5} |  |
| Zelfverzekerd (2) | ZELFVERZEKERD#2 | NUMBER {1 – 5} |  |
| Alert (2) | ALERT#2 | NUMBER {1 – 5} |  |
| Geïnspireerd (2) | GEINSPIREERD#2 | NUMBER {1 – 5} |  |
| Vastberaden (2) | VASTBERADEN#2 | NUMBER {1 – 5} |  |
| Aandacht (2) | AANDACHT#2 | NUMBER {1 – 5} |  |
| Energiek (2) | ENERGIEK#2 | NUMBER {1 – 5} |  |
| Negatieve affect (2) | NA#2 | NUMBER {10 – 50} |  |
| Positieve affect (2) | PA#2 | NUMBER {10 – 50} |  |
|  |  |  |  |
| *PRESENCE (IPQ)* | | | |
| Ik had het gevoel aanwezig te zijn in de computerwereld | GP | NUMBER {1 – 7} | 1 = Helemaal mee oneens  7 = Helemaal mee eens |
| Hoe werkelijk kwam de virtuele wereld op u over | REAL3 | NUMBER {1 – 7} |  |
| Ik was me niet bewust van mijn echte omgeving | INV2 | NUMBER {1 – 7} |  |
| Ik voelde me aanwezig in de virtuele ruimte | SP5 | NUMBER {1 – 7} |  |
| Ik had meer het gevoel bezig te zijn in de virtuele ruimte, dan dat ik het gevoel had iets van buitenaf te bedienen. | SP4 | NUMBER {1 – 7} |  |
| Ik ging volledig op in de virtuele wereld | INV4 | NUMBER {1 – 7} |  |
| Ik had niet het gevoel in de virtuele ruimte aanwezig te zijn | SP3 | NUMBER {1 – 7} |  |
| Ik had het gevoel omgeven te zijn door de virtuel wereld | SP1 | NUMBER {1 – 7} |  |
| Hoe echt kwam de virtuele omgeving op u over | REAL1 | NUMBER {1 – 7} |  |
| Ik lette nog op de echte omgeving | INV3 | NUMBER {1 – 7} |  |
| Ik had het gevoel slechts plaatjes te aanschouwen | SP2 | NUMBER {1 – 7} |  |
| In hoeverre kwam uw ervaring in de virtuele omgeving | REAL2 | NUMBER {1 – 7} |  |
| Hoe bewust was u zich van de echte omgeving, terwijl u zich bevond in de virtuele ruimte | INV1 | NUMBER {1 – 7} |  |
| De virtuele wereld kwam echter op mij over dan de werkelijke wereld | REAL4 | NUMBER {1 – 7} |  |
| SP1 - 1 | SP1u | NUMBER {0 – 6} | Volgens de website van IPQ (  [http://www.igroup.org/ pq/ipq/index.php](http://www.igroup.org/pq/ipq/index.php)) loopt de range van 0 tot en met 6. |
| SP2 - 1 | SP2u | NUMBER {0 – 6} |  |
| SP3 - 1 | SP3u | NUMBER {0 – 6} |  |
| SP4 - 1 | SP4u | NUMBER {0 – 6} |  |
| SP5 - 1 | SP5u | NUMBER {0 – 6} |  |
| INV1 - 1 | INV1u | NUMBER {0 – 6} |  |
| INV2 - 1 | INV2u | NUMBER {0 – 6} |  |
| INV3 - 1 | INV3u | NUMBER {0 – 6} |  |
| INV4 - 1 | INV4u | NUMBER {0 – 6} |  |
| REAL1 - 1 | REAL1u | NUMBER {0 – 6} |  |
| REAL2 - 1 | REAL2u | NUMBER {0 – 6} |  |
| REAL3 - 1 | REAL3u | NUMBER {0 – 6} |  |
| REAL4 - 1 | REAL4u | NUMBER {0 – 6} |  |
| -1 * SP2u +6 | SP2POL | NUMBER {0 – 6} | Ompoolen van items |
| -1 * SP3u +6 | SP3POL | NUMBER {0 – 6} |  |
| -1 * INV3u + 6 | INV3POL | NUMBER {0 – 6} |  |
| -1 * REAL1u + 6 | REAL1POL | NUMBER {0 – 6} |  |
| General presence (GP – 1) | GPu | NUMBER {0 – 6} |  |
| Spatial presence | SP | NUMBER {0 – 6} |  |
| Involvement | INV | NUMBER {0 – 6} |  |
| Experienced realness | REAL | NUMBER {0 – 6} |  |
|  |  |  |  |
| *Vragen over de opdracht (1)* | | | |
| Tijdens het uitvoeren van de taak in de virtuele omgeving dacht ik aan een mogelijke uitvoering ervan in de echter wereld. | DenkenOpdracht1 | NUMBER {-3 – 3} | -3 = Helemaal mee oneens  0 = Neutraal  3 = Helemaal mee eens |
| Ik zou moeite hebben gehad om me te concentreren als ik deze taak later als opdracht in de werkelijkheid had moeten uitvoeren. | Concentratie1 | NUMBER {-3 – 3} |  |
| Ik denk dat ik de taak in de werkelijkheid goed zou uitvoeren | UitvoerenTaak1 | NUMBER {-3 – 3} |  |
| Ik zou er tegen op zien om de opdracht in de werkelijkheid uit te voeren. | UitvoerenWerkelijkheid1 | NUMBER {-3 – 3} |  |
| Deze virtuele taak zou me goed voorbereiden op een zelfde taak in de echte wereld. | VoorbereidenWerkelijkheid1 | NUMBER {-3 – 3} |  |
| Geef op de schaal (van 0 tot en met 10) aan hoe goed u de taak in de echte wereld denkt te kunnen volbrengen. | UitvoerenCijfer1 | NUMBER {0 – 10} |  |
| Welke problemen heeft u in gedachte komen bij het uitvoeren van dezelfde taak in de echte wereld? | ProblemenUitvoeren1 | TEXT |  |
| *Vragen over de opdracht (2)* | | | |
| Tijdens het uitvoeren van de taak in de virtuele omgeving dacht ik de hele tijd aan de opdracht in de werkelijkheid. | DenkenOpdracht2 | NUMBER {-3 – 3} | -3 = Helemaal mee oneens  0 = Neutraal  3 = Helemaal mee eens |
| Ik had moeite me te concentreren op de taak. | Concentratie2 | NUMBER {-3 – 3} |  |
| Ik denk dat ik de taak in de werkelijkheid goed kan uitvoeren. | UitvoerenTaak2 | NUMBER {-3 – 3} |  |
| Ik zie er tegen op om de opdracht in de werkelijkheid uit te voeren. | UitvoerenWerkelijkheid2 | NUMBER {-3 – 3} |  |
| Deze virtuele taak heeft me goed voorbereid op de taak in de echte wereld. | VoorbereidenWerkelijkheid2 | NUMBER {-3 – 3} |  |
| Geef op de schaal (van 0 tot en met 10) aan hoe goed u denkt de taak in de echte wereld te zullen volbrengen. | UitvoerenCijfer2 | NUMBER {0 – 10} |  |
| Welke problemen denkt u te zullen tegenkomen bij het uitvoeren van dezelfde taak in de echte wereld? | ProblemenUitvoeren2 | TEXT |  |
|  |  |  |  |
| *Navigatie* | | | |
| Moest u uw aandacht gebruiken voor de navigatie? | NavigatieAandacht | NUMBER {1 – 5} | 1 = Helemaal niet of nauwelijks  2 = Een beetje  3 = Matig  4 = Veel  5 = Heel veel |
| Hinderde de besturing u bij het uitvoeren van de opdracht in de virtuele omgeving? | NavigatieHinderen | NUMBER {1 – 5} |  |
| *Overige* | | | |
| Heeft u nog opmerkingen over dit experiment? | OPMERKINGEN | TEXT |  |
| Misselijk |  | NUMBER {0 – 1} | 0 = NEE (niet gemeld)  1 = JA |
