# Supplementary material for: Effects of personal relevance and simulated darkness on the affective appraisal of a virtual environment
Source: PeerJ. 2016 Feb 25;4:e1743. doi: 10.7717/peerj.1743 (PMC4788201; doi:10.7717/peerj.1743)
Supplement: Supplemental Information 1 — Full listing of all measures used in the study. [file peerj-04-1743-s002.doc]

Measures

This document presents the questionnaires that were used to measure respectively

- the participants’ affective appraisal of the VE,
- their fear of darkness in real life,
- their emotional state,
- their emotional response to the VE and to the follow-up task,
- their sense of presence in the VE, and
- their game and navigation experience.

Contents

[Environmental appraisal scale 2](#__RefHeading___Toc437433430)

[Fear of darkness in the real world 3](#__RefHeading___Toc437433431)

[Self-Assessment Manikin (SAM) 4](#__RefHeading___Toc437433432)

[Positive and Negative Affect Scale (PANAS ) 5](#__RefHeading___Toc437433433)

[Igroup Presence Questionnaire (IPQ) 7](#__RefHeading___Toc437433434)

[Game and navigation experience 9](#__RefHeading___Toc437433435)

[REFERENCES 10](#__RefHeading___Toc437433436)

# Environmental appraisal scale

The affective appraisal of the VE was measured using a subset of the 38 adjectives from a differential rating scale that was designed to assess the atmosphere of built environments (Vogels, 2008a). In this context atmosphere is defined as the affective evaluation of the environment. Atmosphere gives information about the expected effect of the environment on people’s affective state (Vogels, 2008b). The 11 selected terms represent each of its four principal affective dimensions (Vogels, 2008a):

**Environmental appraisal scale**

| Affective dimension | Dutch term | English translation |
| --- | --- | --- |
| *Cosiness* | *behaaglijk* | *cosy* |
|  | *intiem* | *intimate* |
|  | *veilig* | *safe* |
| *Tenseness* | *gespannen* | *tense* |
|  | *beangstigend* | *terrifying* |
|  | *bedreigend* | *threatening* |
| *Detachment* | *zakelijk* | *business* |
|  | *formeel* | *formal* |

Each term was scored on a 7-point rating scale (-3 = *not at all*, 3= *very much*).

# Fear of darkness in the real world

In the real world cues like darkness (day/night), novelty (familiar/unfamiliar) and lack of social presence are known to evoke fear of victimization and determine navigation behavior (Fisher & Nasar, 1992; Warr, 1984; Warr, 1990). We used the following statements to assess the susceptibility of our participants to each of these cues:

1. *I’m very well able to find my way in an unfamiliar environment.*
2. *I’m very well able to find my way in a familiar environment at night.*
3. *I’m very well able to find my way in an unfamiliar environment at night.*
4. *I can orientate very well in the dark.*
5. *I can orientate very well in daytime.*
6. *I dare to walk by myself in an unfamiliar environment at night.*
7. *I dare to walk by myself in an unfamiliar environment in daytime.*
8. *I feel uncomfortable in the dar*k.

Participants scored the extent to which they agreed with each of these statements on a 7-point bipolar rating scale (-3 = *strongly disagree*, 3= *strongly agree*).

# Self-Assessment Manikin (SAM)

The participants self-reported their momentary feelings of pleasure, arousal and dominance using a validated 9-point pictorial rating scale (the Self-Assessment Manikin or SAM: Bradley & Lang, 1994). The SAM provides a simple, fast, and non-linguistic way of assessing emotional state along the principal emotional dimensions

of Valence (Pleasure), Arousal and Dominance. SAM scales can be downloaded from <http://irtel.uni-mannheim.de/pxlab/demos/index_SAM.html>.

**SAM Scales**


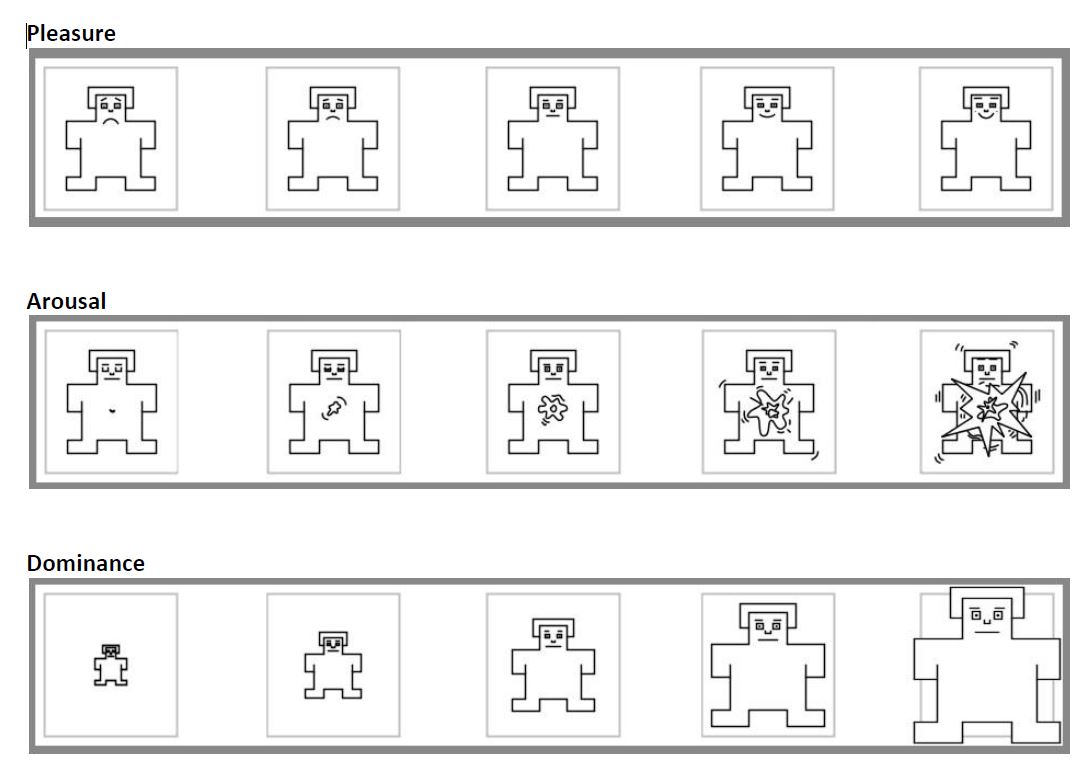


# Positive and Negative Affect Scale (PANAS )

In this study Emotional State was measured through self-assessment using a Dutch translation of the Positive and Negative Affect Scale (PANAS: Watson, Clark & Tellegen, 1988). We based our scale on two validated translations: one by Peters et al. (Peeters, Ponds & Vermeeren, 1996) and one by Engelen (Engelen et al., 2006). Although there are slight differences it has been observed that both translations are equivalent (Engelen et al., 2006). We selected the translation by Engelen et al. (2006) where we replaced 4 items by corresponding items from the translation of Peters et al. (1996) since they were slightly more appropriate in the present context.

The PANAS is a list of 20 adjectives used to describe different emotional states:

- 10 states of Positive Affect (PA) and
- 10 states of Negative Affect (NA).

The PA scale measures activity and pleasure, while the NA scale relates to fear and stress.

Because of its length (and in contrast to the SAM) the PANAS is more suitable to measure longer lasting emotional states.

Participants scored the extent to which they experienced each emotional state on a 5-point unipolar rating scale:

1 = *not at all or very slightly,*

*2 = a little*

*3 = moderately*

*4 = a lot*

*5 = extremely*.

**PANAS Scale**

|  | **Original PANAS items  (Watson et al., 1988)** | **Dutch translation PANAS items** |
| --- | --- | --- |
| **NA1** | Distressed | Bedroefd^1^ |
| **NA2** | Upset | Terneergeslagen^1^ |
| **NA3** | Guilty | Schuldig^1^ |
| **NA4** | Scared | Angstig^1^ |
| **NA5** | Hostile | Vijandig^1^ |
| **NA6** | Irritable | Prikkelbaar^2^ |
| **NA7** | Ashamed | Beschaamd^1^ |
| **NA8** | Nervous | Nerveus^2^ |
| **NA9** | Jittery | Rusteloos^2^ |
| **NA10** | Afraid | Bang^1^ |
|  |  |  |
| **PA1** | Interested | Geïnteresseerd^1^ |
| **PA 2** | Excited | Opgewekt^1^ |
| **PA 3** | Strong | Sterk^1^ |
| **PA 4** | Enthusiastic | Enthousiast^1^ |
| **PA 5** | Proud | Zelfverzekerd^1^ |
| **PA 6** | Alert | Alert^1^ |
| **PA 7** | Inspired | Geïnspireerd^2^ |
| **PA 8** | Determined | Vastberaden^1^ |
| **PA 9** | Attentive | Aandachtig^1^ |
| **PA 10** | Active | Energiek^1^ |

^1^ = from Engelen et al., 2006

^2^ = from Peeters et al, 2006

# Igroup Presence Questionnaire (IPQ)

In this study we used the Dutch translation of the Igroup Presence Questionnaire (IPQ) which can be downloaded from <http://www.igroup.org/pq/ipq>; see Schubert, Friedmann & Regenbrecht, 2001).

The IPQ contains 14 questions addressing the factors

- *General Presence* (GPR; item 1),
- *Spatial Presence* (SPR; items 2-6),
- *Involvement* (INV; items 7-10) and
- *Realism* (REA; items 11-14).

Each question is scored on a bipolar 7-point rating scale (ranging from -3 to 3).

- The factor GPR measures the general sensation of actually being in the VE.
- The factor SPR addresses the spatial aspects of the VE experience.
- The factor INV measures to what degree the participant’s attention was absorbed by the VE.
- The factor REA measures the extent to which the VE was perceived as realistic.

**Igroup Presence Questionnaire**

| **Nr** | **Factor** | **Question** | **Range** |
| --- | --- | --- | --- |
| 1 | GPR | In the virtual environment I had a sense of ‘being there’. | not at all –  very much |
| 2 | SPR | Somehow I felt that the virtual world surrounded me. | fully disagree –fully agree |
| 3 | SPR | I felt like I just perceived pictures. | fully disagree –fully agree |
| 4 | SPR | I did not feel present in the virtual space. | did not feel –  felt present |
| 5 | SPR | I had a sense of acting in the virtual space, rather than operating something from outside. | fully disagree –fully agree |
| 6 | SPR | I felt present in the virtual space. | fully disagree –fully agree |
| 7 | INV | How aware were you of the real world surrounding while navigating in the virtual world? (i.e., sounds, room temperature, other people, etc.)? | extremely aware-moderately aware-not aware at all |
| 8 | INV | I was not aware of my real environment. | fully disagree –fully agree |
| 9 | INV | I still paid attention to the real environment. | fully disagree –fully agree |
| 10 | INV | I was completely captivated by the virtual world. | fully disagree –fully agree |
| 11 | REA | How real did the virtual world seem to you? | completely real –not real at all |
| 12 | REA | How much did your experience in the virtual environment seem consistent with your real world experience? | not consistent –moderately consistent –  very consistent |
| 13 | REA | How real did the virtual world seem to you? | about as real as an imagined world –indistinguishable from the real world |
| 14 | REA | The virtual world seemed more realistic than the real world. | fully disagree –fully agree |

# Game and navigation experience

Game experience was measured by the following two questions

1. *How frequently do you play 3D computer games?*
2. *How frequently do you use other virtual environments (e.g., Second Life)?*

and scored on a 5-point unipolar rating scale (1=*never, 5= very often*).

The extent to which navigation in the present simulation required attention and interfered with task performance was measured by the following two questions:

1. *Did you need your attention to navigate*?
2. *Did the navigation control hinder your task performance in the virtual environment*?

and scored on a 5-point unipolar rating scale (1= *not at all, 5= very much*).

# REFERENCES

Bradley, M.M. & Lang, P.J. 1994. Measuring emotion: the self-assessment manikin and the semantic differential. *Journal of Behavior Therapy and Experimental Psychiatry, 25(1),* 49-59. DOI 10.1016/0005-7916(94)90063-9.

Engelen, U., De Peuter, S., Victoir, A., Van Diest, I. & Van den Bergh, O. 2006. Verdere validering van de Positive and Negative Affect Schedule (PANAS) en vergelijking van twee Nederlandstalige versies [Further validation of the Positive and Negative Affect Schedule (PANAS) and comparison of two Dutch versions.]. *Gedrag & Gezondheid, 34(2),* 61-70. DOI 10.1007/BF03087979.

Fisher, B.S. & Nasar, J.L. 1992. Fear of crime in relation to three exterior site features: Prospect, refuge, and escape. *Environment and Behavior, 24(1),* 35-65.

IJsselsteijn, W.A., de Ridder, H., Freeman, J. & Avons, S.E. 2000. Presence: concept, determinants and measurement. *Human vision and electronic imaging V*, SPIE-3959 (pp. 520-529). Bellingham, WA, USA: Society of Photo-Optical Instrumentation Engineers. DOI 10.1117/12.387188.

Peeters, F.P.M.L., Ponds, R.W.H.M. & Vermeeren, M.T.G. 1996. Affectiviteit en zelfbeoordeling van depressie en angst (Affectivity and self-assessment of depression and fear). *Tijdschrift voor Psychiatrie, 38(3),* 240-250.

Schubert, T., Friedmann, F. & Regenbrecht, H. 2001. The experience of presence: factor analytic insights. *Presence: Tele-operators and Virtual environments, 10(3),* 266-281. DOI 10.1162/105474601300343603.

Vogels, I. 2008a. Atmosphere metrics. Development of a tool to quantify experienced atmosphere. In J.H.D.M. Westerink, M. Ouwerkerk, T.J.M. Overbeek, F. Pasveer & B. de Ruyter (Eds.), *Probing experience. From assessment of user emotions and behaviour to development of products*, Philips Research Book Series 8 (pp. 25-41). Dordrecht, The Netherlands: Springer Netherlands.

Vogels, I. 2008b. Atmosphere metrics: a tool to quantify perceived atmosphere. *International Symposium "Creating an Atmosphere"*, http://www.cresson.archi.fr/AMBIANCE2008-commS1.htm#s1IV, last accessed 4-10-2011 (pp. 1-6). Grenoble, France: CRESSON.

Warr, M. 1984. Fear of victimization: why are women and the elderly more afraid? *Social Science Quarterly, 65(3),* 681-702.

Warr, M. 1990. Dangerous situations: social context and fear of victimization. *Social Forces, 68(3),* 891-907. DOI 10.1093/sf/68.3.891.

Watson, D., Clark, L.A. & Tellegen, A. 1988. Development and validation of brief measures of positive and negative affect: the PANAS scales. *Journal of Personality and Social Psychology, 54(6),* 1063-1070. DOI 10.1037/0022-3514.54.6.1063.
